# TANGO1 and SEC12 are copackaged with procollagen I to facilitate the generation of large COPII carriers

**DOI:** 10.1073/pnas.1814810115

**Published:** 2018-12-13

**Authors:** Lin Yuan, Samuel J. Kenny, Juliet Hemmati, Ke Xu, Randy Schekman

**Affiliations:** ^a^Department of Molecular and Cell Biology, University of California, Berkeley, CA 94720;; ^b^Howard Hughes Medical Institute, University of California, Berkeley, CA 94720;; ^c^Department of Chemistry, University of California, Berkeley, CA 94720;; ^d^Chan Zuckerberg Biohub, San Francisco, CA 94158

**Keywords:** COPII, collagen, SEC12, TANGO1, secretion

## Abstract

Large coat protein complex II (COPII)-coated vesicles serve to convey the large cargo procollagen I (PC1) from the endoplasmic reticulum (ER). The link between large cargo in the lumen of the ER and modulation of the COPII machinery remains unresolved. TANGO1 is required for PC secretion and interacts with PC and COPII on opposite sides of the ER membrane, but evidence suggests that TANGO1 is retained in the ER, and not included in normal size (<100 nm) COPII vesicles. Here we show that TANGO1 is exported out of the ER in large COPII-coated PC1 carriers, and retrieved back to the ER by the retrograde coat, COPI, mediated by the C-terminal RDEL retrieval sequence of HSP47. TANGO1 is known to target the COPII initiation factor SEC12 to ER exit sites through an interacting protein, cTAGE5. SEC12 is important for the growth of COPII vesicles, but it is not sorted into small budded vesicles. We found both cTAGE5 and SEC12 were exported with TANGO1 in large COPII carriers. In contrast to its exclusion from small transport vesicles, SEC12 was particularly enriched around ER membranes and large COPII carriers that contained PC1. We constructed a split GFP system to recapitulate the targeting of SEC12 to PC1 via the luminal domain of TANGO1. The minimal targeting system enriched SEC12 around PC1 and generated large PC1 carriers. We conclude that TANGO1, cTAGE5, and SEC12 are copacked with PC1 into COPII carriers to increase the size of COPII, thus ensuring the capture of large cargo.

## Significance

Collagen is a major component of the extracellular matrix, and its secretion requires cytoplasmic proteins that assemble on the surface of the endoplasmic reticulum to bud ∼100-nm-diameter cargo transport vesicles (COPII). Bulky collagens, such as the 300-nm procollagen I (PC1), are too big to fit into normal COPII vesicles. Recently, large COPII-coated vesicles were found to act as PC1 carriers, but how these large COPII carriers are generated remains unclear. Here, we show copackaging of PC1 along with its cargo receptor TANGO1, a coreceptor protein, cTAGE5, and the COPII initiating factor SEC12. Because SEC12 is excluded from small COPII vesicles, we propose that TANGO1 targets SEC12 to PC1-containing endoplasmic reticulum and drives the formation of large COPII-coated vesicles.

The coat protein complex II (COPII) is required for the endoplasmic reticulum (ER) export of most secretory proteins and transmembrane proteins destined for the plasma membrane (1). COPII subunits cooperate to generate transport vesicles that carry secretory cargos to the Golgi apparatus (2). The ER membrane protein SEC12 recruits and activates the small GTPase SAR1 by catalyzing a GDP to GTP exchange (3). SAR1-GTP extends an N-terminal amphipathic helix that embeds in the ER membrane to initiate a vesicle bud (4). SAR1-GTP also recruits the inner layer of COPII coat proteins SEC23/24, which in turn recruit the outer layer COPII coat proteins SEC13/31. Assembled COPII envelops a membrane bud and, on vesicle fission, the coat is shed by the acceleration of GTP hydrolysis by SAR1 (5).

COPII-coated vesicles are usually observed as small vesicles with diameters under 100 nm, of sufficient size to transport most secretory cargos (2, 6, 7). Some secretory cargos, such as the 300-nm-long procollagen I (PC1) rigid rod, require COPII for their secretion, but are seemingly too large to be accommodated by conventional COPII-coated vesicles (8–10). Recently, we reported the existence of large COPII-coated PC1 carriers with diameters above 300 nm visualized using correlated light electron microscopy (CLEM), stochastic optical reconstruction microscopy (STORM), and live-cell imaging in multiple PC1-secreting cultured human cell lines (11). Although the formation of large ER transport vesicles is promoted by monoubiquitylation of the large subunit of the outer coat, SEC31A (12), the molecular link between ubiquitylation and the change in COPII polymerization is not understood.

**Fig. 1.**
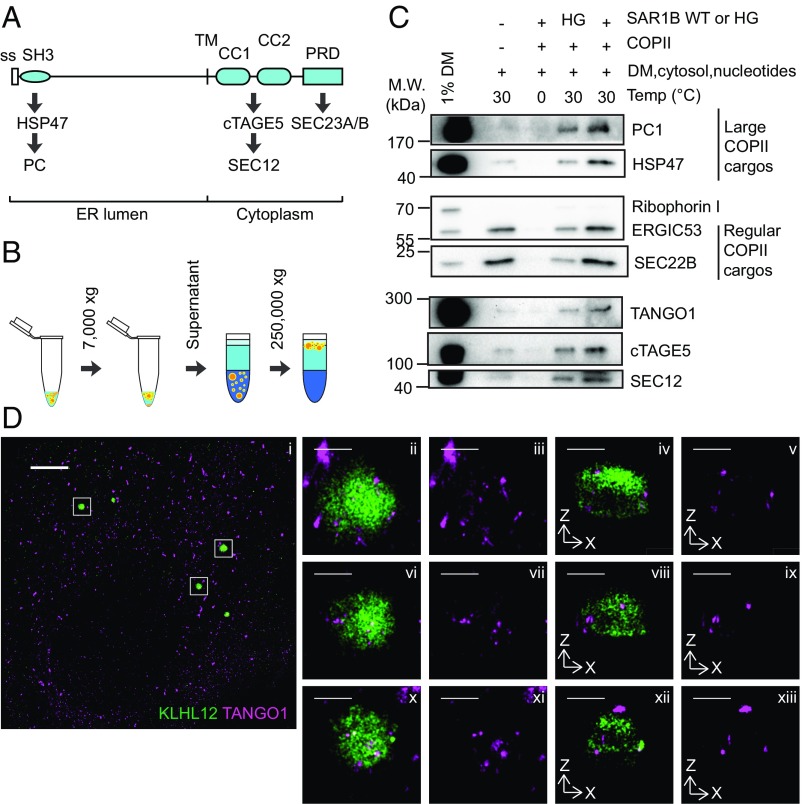
TANGO1 is copackaged with PC1 into large COPII carriers. (*A*) Schematic representation of TANGO1’s domain structure with information on relevant interactions. The N-terminal SH3 domain interacts with the PC-specific chaperone HSP47, which binds to folded PCs (16). The coiled-coil (CC) domains of TANGO1 form a stable complex with cTAGE5 and SEC12 (17–19). The PRD of TANGO1 interacts with the COPII inner coat protein SEC23 (14, 20). (*B*) Scheme depicting the isolation of COPII carriers from cell-free budding reactions as previously described (11, 22). Briefly, COPII vesicles were generated by incubating a reaction containing donor membrane prepared from IMR-90 cells, purified recombinant COPII proteins (1 μg of SAR1B wild-type or H79G, 1 μg of SEC23A/24D, 1 μg of SEC13/31A), nucleotides, and 2 μg/μL HT1080 cytosol at 30 °C for 1 h. Vesicles in 7,000 × *g* supernatant fractions from budding reactions were isolated by flotation. (*C*) TANGO1, cTAGE5, and SEC12 in COPII carriers were detected by immunoblotting aliquots of the top float fractions of reactions conducted under different conditions. Donor membrane (DM) was included as an input control. PC1 and HSP47 are captured into large COPII-coated PC1 carriers and serve as positive controls for large COPII carriers. ERGIC53 and SEC22B are found in conventional COPII vesicles and serve as further controls for COPII vesicles. Ribophorin I is an ER resident protein that serves as a negative control. (*D*) Dual color 3D-STORM images of a KI6 cell containing multiple large COPII coated vesicles. Overview of the cell is shown in (*i*), and *Insets* are enlarged and shown in (*ii*–*xiii*). TANGO1 (magenta) colocalized with KLHL12-FLAG (green) which coats large COPII vesicles in *xy* maximum projections (*ii*, *iii*, *vi*, *vii*, *x*, *xi*) or respective *xz* cross-sections (*iv*, *v*, *viii*, *ix*, *xii*, *xiii*). (Scale bars: 5 μm in *D*, *i*; 500 nm in *D*, *ii*–*xiii*.)

Secretory proteins are collected into nascent COPII buds through the intervention of a membrane sorting receptor (13). One such receptor, TANGO1 (MIA3), is required for PC secretion through its interaction with PCs in the ER lumen and the SEC23/24 subunits on the cytoplasmic surface of the ER (14, 15). The luminal SH3 domain of TANGO1 interacts with the PC-specific chaperone HSP47, which accompanies folded PCs to the *cis*-Golgi or the ER-Golgi intermediate compartment (ERGIC) in large COPII carriers (Fig. 1*A*) (11, 14, 16). TANGO1 also forms a stable complex with two other transmembrane proteins: cTAGE5 and the COPII initiating factor, SEC12 (17–19) (Fig. 1*A*). The cytosolic proline-rich domains (PRD) of both TANGO1 and cTAGE5 also interact directly with the COPII inner coat protein, SEC23 (14, 20) (Fig. 1*A*). Therefore, TANGO1 has the molecular features of a COPII receptor for large cargo.

Cargo receptors, such as ERGIC53 (LMAN1 or p58), are efficiently sorted into COPII vesicles for anterograde trafficking and are then recycled back to the ER in COPI vesicles (13). In a living cell, newly generated COPII vesicles are efficiently targeted for fusion with their destination organelles, making it challenging to isolate and characterize vesicle cargo proteins. Fortunately, COPII-coated vesicles can be generated from purified components in a cell-free reaction and thus are more easily isolated; this has proven to be a powerful means to detect the incorporation of cargo receptors, such as ERGIC53 and Erv29p (6, 21). Although this reaction typically generates small COPII vesicles, we recently developed an alternative budding and isolation protocol to detect the capture of PC1 into large COPII-coated carriers, which were evaluated by immunoblotting, structured illumination microscopy, flow cytometry, and thin-section transmission electron microscopy (11, 22).

Here, we examine large COPII-coated PC1 carriers generated in a cell-free reaction and observe that TANGO1, cTAGE5, and SEC12 are copacked with PC1. TANGO1 and SEC12 were also observed on endogenous large COPII-coated PC1 carriers by 3D-STORM superresolution microscopy. Exported TANGO1 is recycled back to the ER with HSP47, a process dependent on the COPI coat, consistent with the typical itinerary of a COPII cargo sorting receptor. In contrast to the exclusion of SEC12 from regular COPII vesicles, SEC12 was enriched around PC1 in budding membrane profiles. To test the effect of actively targeting SEC12 to large cargo by TANGO1, we reconstituted the targeting with minimal components in cultured cells and observed the formation of large PC1 carriers containing SEC12. Thus, our data reveal a mechanism in which the large cargo receptor, TANGO1, coordinates the formation of large COPII carriers by actively targeting SEC12 to ER exit sites engaged in the capture of large cargo.

## Results

### TANGO1, cTAGE5, and SEC12 Are Copackaged into Large COPII Carriers Along with PC1.

The large transmembrane protein TANGO1 is poised to be a COPII receptor for large PC cargo as it interacts with COPII and PC on opposite sides of the ER membrane (Fig. 1*A*) (14, 16, 18–20). To test whether TANGO1 is incorporated into COPII carriers with large cargos, we devised a cell-free reaction to generate large COPII-coated PC1 carriers from purified components (11, 22). Following the completion of the reaction, an alternative purification method was used, where donor membrane was sedimented in a 10-min centrifugation at 7,000 × *g*, and vesicles in the supernatant were separated from soluble components by buoyant density flotation in a step gradient (Fig. 1*B*).

Using this isolation method, we previously demonstrated that the capture of PC1 into large COPII-coated membrane carriers was dependent on the presence of COPII coat proteins as well as GTP hydrolysis by the COPII subunit SAR1 (11). Consistent with our previous report, the large cargo PC1, the collagen-specific chaperone HSP47, and the control COPII cargos ERGIC53 and SEC22B were observed in the floated fraction produced in a reaction containing membranes, COPII, and nucleotide (Fig. 1*C*). Cargo capture was dependent on COPII, and it was reduced in an incubation containing a GTPase mutant, SAR1 H79G (Fig. 1*C*). Thus, the COPII-dependent generation of PC1 carriers was recapitulated in the cell-free assay.

Under optimal conditions of temperature and recombinant COPII proteins, all three components of the TANGO1/cTAGE5/SEC12 complex were detected by immunoblotting in the floated fraction (19) (Fig. 1 *A* and *C*). The amount of TANGO1, cTAGE5, and SEC12 detected in the floated fraction decreased when recombinant COPII was omitted or when the SAR1B H79G mutant was used in place of wild-type SAR1B. Notably, the export of PC1 and proteins implicated in PC1 secretion—namely HSP47, TANGO1, cTAGE5, and SEC12—showed a higher dependency on recombinant COPII compared with cargos of conventional small COPII vesicles, as more dramatic decreases were observed in PC1, HSP47, TANGO1, cTAGE5, and SEC12 when recombinant COPII was omitted from the reaction (Fig. 1*C*, compare lanes 2 from the left to the rightmost lanes). Taken together, these results show the export of TANGO1, cTAGE5, and SEC12 require GTP hydrolysis by the COPII subunit SAR1 and the generation of COPII-coated vesicles.

Previous work has shown that mild overexpression of KLHL12, a substrate adaptor of the cullin 3 (CUL3) ubiquitin ligase, leads to the formation of large COPII vesicles and an enhanced rate of traffic of PC1 from the ER to the Golgi complex (11, 12). We engineered human cells (KI6) that overexpress KLHL12 under doxycycline-controlled transcriptional activation and found 7.5 h of induction was optimal to observe large PC1-containing COPII structures (11). These large COPII structures were resolved by 3D-STORM, revealing hollow spheres of KLHL12 and the COPII coat protein SEC31A that encapsulated the large cargo PC1 (11). We also visualized endogenous large COPII-coated PC1 carriers in Saos-2 cells using 3D-STORM and showed SEC31A and endogenous KLHL12 enveloping endogenous PC1 (11). KI6 and Saos-2 cells were used interchangeably in this study, and both SEC31A and KLHL12 were used as markers for large COPII carriers. To test whether TANGO1 was incorporated into large COPII carriers in cells, we performed immunofluorescence labeling of TANGO1 and KLHL12 in KI6 cells induced for 7.5 h, as before. Large, hollow spheres of KLHL12 >300 nm in diameter were observed, as previously reported (11), and TANGO1 was observed on these large COPII carriers (Fig. 1*D*). The superresolution visualization and cell-free biochemical results confirm each other.

### TANGO1 Is Recycled with HSP47 by a COPI-Dependent Process.

**Fig. 2.**
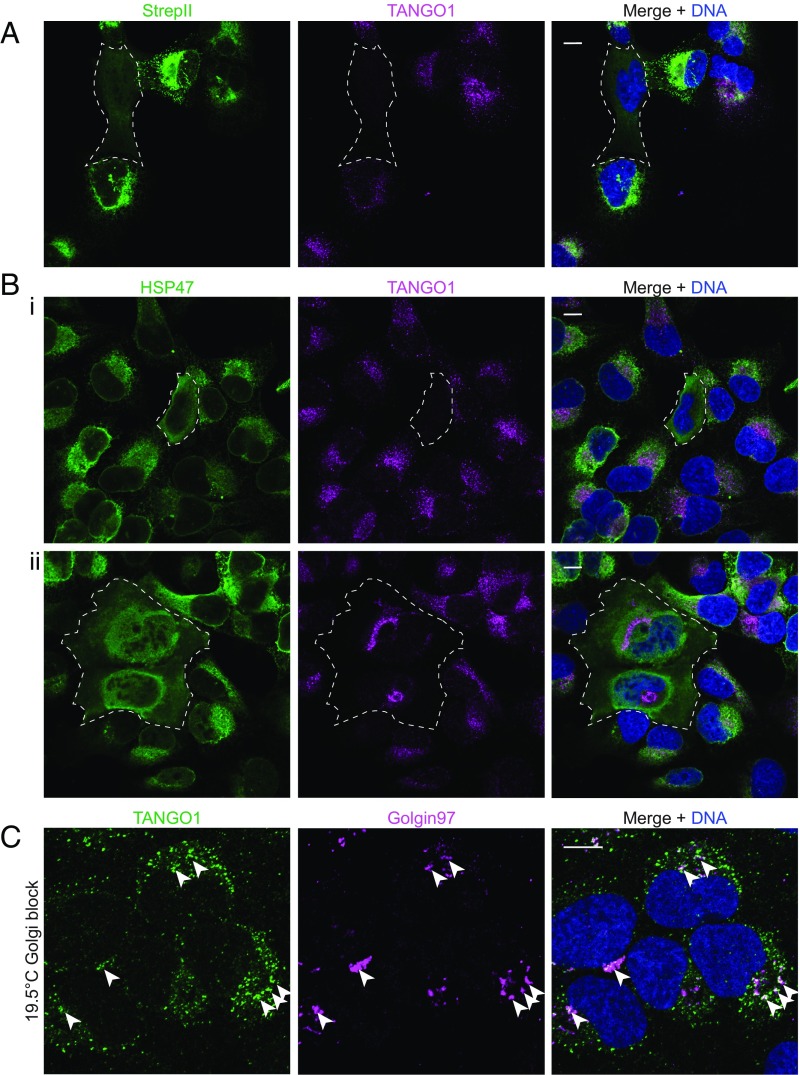
TANGO1 is retrieved with HSP47 via its c-terminal RDEL motif. Confocal microscopy images of U-2OS cells transfected with StrepII-HSP47ΔRDEL. (*A* and *B*) Immunofluorescent labeling using antibodies against the StrepII tag (*A*; green) or HSP47 (*B*; green) and TANGO1 (magenta). In wild-type cells, TANGO1 localized at ERES and HSP47 localized in the ER (Fig. 3*A*). TANGO1 mis-localization is observed following the overexpression and secretion of StrepII-HSP47ΔRDEL in cells marked inside dotted lines. In these cells, HSP47 (green) no longer shows ER localization, but rather appeared to localize at the cell surface; little TANGO1 (magenta) is detected in most cells (*A* and *B*, *i*), and localizes to the Golgi membrane in some cells (*B*, *ii*). (*C*) Cells transfected with StrepII-HSP47ΔRDEL were incubated at 19.5 °C in the presence of ascorbate for 3 h to accumulate cargo in the Golgi and followed by immunofluorescence labeling targeting of TANGO1 (green) and a Golgi marker, Golgin97 (magenta). Arrowheads point to examples of TANGO1 colocalized with Golgin97. (Scale bars, 10 μm.)

Similar to other cargo receptors like ERGIC53, TANGO1 localizes at ER exit sites (ERES) in cells at steady state, such that it significantly colocalizes with ERGIC53 and occasionally colocalizes with the *cis*-Golgi marker GM130 (*SI Appendix*, Fig. S1). Unlike most cargo adaptors, TANGO1 does not contain a C-terminal KKXX or KDEL retrieval signal. Alternatively, the collagen chaperone HSP47 may be responsible for retrieval of this receptor as it interacts with the C-terminal SH3 domain of TANGO1 (16). HSP47 recognizes the folded triple-helical domain of PCs in the ER (23), and serves as a chaperone to convey cargo in large COPII carriers to the ERGIC or *cis*-Golgi compartment (11). Subsequently, cargo is released due to the lower pH in the lumen of ERGIC or *cis*-Golgi, and HSP47 is recycled back to the ER via its C-terminal RDEL sequence (24, 25). Efficient recycling results in the steady-state localization of HSP47 to the ER. When the C-terminal RDEL sequence is deleted, HSP47ΔRDEL is readily secreted (24). To test whether TANGO1 is retrieved via its interaction with HSP47, we overexpressed a StrepII-tagged HSP47ΔRDEL and observed that in cells that had secreted this fusion, TANGO1 either became undetectable (Fig. 2 *A* and *B*, *i*) or mislocalized to the Golgi apparatus (Fig. 2 *B*, *ii*). It is possible that TANGO1 trafficked to the lysosome from the Golgi due to failed retrieval, resulting in its failure to be detected. To test this possibility, we incubated cells that overexpressed HSP47ΔRDEL at 19.5 °C for 3 h to accumulate cargo in the Golgi apparatus, and observed colocalization of TANGO1 with a *trans*-Golgi network marker Golgin97 (Fig. 2*C*). Because TANGO1 was not observed beyond ERGIC and *cis*-Golgi at steady state (*SI Appendix*, Fig. S1), the detection of TANGO1 in the *trans*-Golgi network was likely a result of inefficient retrieval from the ERGIC and *cis*-Golgi. These results suggest that the interaction between TANGO1 and HSP47 influences the retrieval of TANGO1.

**Fig. 3.**
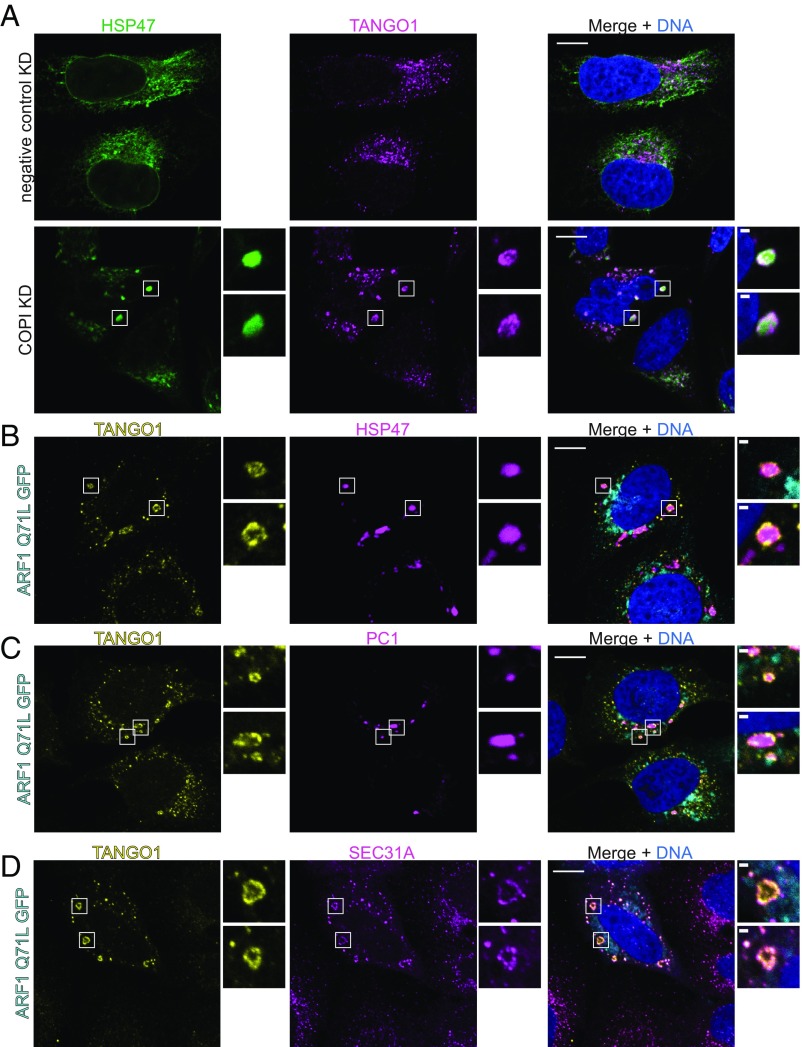
TANGO1 localizes around giant COPII membranes in cells depleted of COPI. (*A*) Confocal images of U-2os-wt-c11 cells transfected with negative control siRNA or siRNA that targeted coatomer subunit δ (*ARCN1* gene) for 48 h followed by immunofluorescence labeling of TANGO1 (magenta) and HSP47 (green). TANGO1 was observed around large HSP47 puncta in cells depleted of COPI. Magnified *Insets* show two examples of such structures. (*B*–*D*) Confocal images of U-2os-wt-c11 cells expressing ARF1 Q71L GFP (cyan) were labeled by immunofluorescence targeting of TANGO1 (yellow) and HSP47 (magenta) in *B* or PC1 (magenta) in *C* or SEC31A (magenta) in *D*. (Scale bars: 10 μm in overviews and 1 μm in magnified *Insets*.)

To test whether TANGO1 is recycled back to the ER by COPI, we depleted COPI in cells with small-interfering RNA (siRNA) that targets coatomer subunit δ (*ARCN1* gene) (26). In cells that were depleted of COPI, we observed accumulation of TANGO1 around concentrated HSP47 structures (Fig. 3*A*). This localization was not observed in cells transfected with negative control siRNA, showing that this phenotype was due to COPI depletion (Fig. 3*A*). Alternatively, COPI trafficking is blocked by overexpressing a GTP-locked ARF1 Q71L mutant (26). In cells that expressed ARF1 Q71L-GFP, TANGO1 accumulated around HSP47 puncta similar to the phenotype observed with siRNA knockdown of coatomer subunit δ (Fig. 3*B*). Taken together, these data suggest that the retrieval of TANGO1 depends on ARF1-GTP hydrolysis and COPI budding.

We further characterized the compartment where TANGO1 accumulated in cells depleted of COPI. TANGO1-decorated HSP47 puncta appeared not to colocalize with ARF1 Q71L-GFP, which localizes in the ERGIC (26). Instead, TANGO1 accumulated around PC1 puncta and colocalized with the COPII outer coat protein SEC31A (Fig. 3 *C* and *D*). These exceptionally large COPII-decorated membranes were much bigger than the functional carriers we observed by STORM and CLEM (11), and were readily resolved by confocal microscopy.

### SEC12 Is Enriched in Large COPII-Coated PC1 Carriers.

We were particularly intrigued by the detection of SEC12 in COPII carriers in our cell-free reaction (Fig. 1*C*), because this protein is not normally sorted into small COPII vesicles (2, 3). A recent study reconstituted the cytosolic and transmembrane domains of the yeast Sec12p and the transmembrane COPII cargo Bet1p on a thick planar lipid bilayer that allowed collection of cargo molecules into curved membrane buds but did not support vesicle scission (27). When COPII proteins (Sar1p, Sec23p/24p, Sec13p/31p) and GTP were supplemented to the planar lipid bilayer containing Sec12p and Bet1p, COPII coat proteins (Sec23p/24p, Sec13p/31p) polymerized into clusters with the cargo Bet1p, resembling prebudding complexes at the ERES (27). Consistent with our own earlier results using native ER membranes as a template for vesicle budding (2), Sec12p was excluded from the reconstituted COPII-cargo clusters, suggesting that it is intrinsically excluded from regular COPII prebudding complexes and thus regular COPII vesicles (27).

**Fig. 4.**
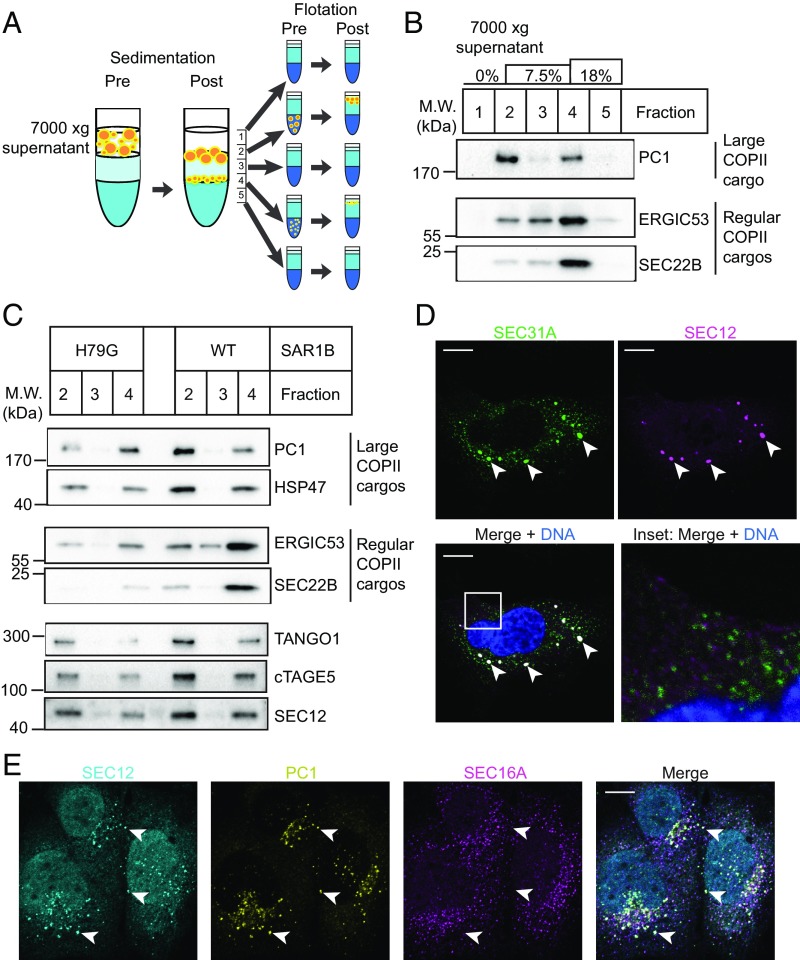
SEC12 is enriched in large COPII-coated PC1 carriers. (*A*) Schematic representation of the fractionation procedure used to separate small and large COPII carriers. Supernatant after 7,000 × *g* centrifugation from a vesicle-budding reaction was overlaid onto a step gradient consisting of 7.5% and 18% OptiPrep. The gradient was centrifuged at 250,000 × *g* for 1 h to separate large from regular cargo-containing COPII carriers. Fractions (numbered 1–5) taken after sedimentation were used as inputs for flotation to separate vesicles from soluble contents. (*B*) Analysis of the large cargo PC1 and regular COPII cargos ERGIC53 and SEC22B in buoyant membrane collected from sedimentation fractions postflotation. (*C*) Budding reactions were supplemented with wild-type or H79G mutant SAR1B. Buoyant membrane from relevant sedimentation fractions were immunoblotted for TANGO1, cTAGE5, and SEC12. PC1 and HSP47 serve as markers for COPII-coated PC1 carriers. ERGIC53 and SEC22B serve as markers for regular COPII vesicles. (*D*) Confocal image of KI6 cells that were induced for KLHL12 overexpression for 7.5 h and immunolabeled with a fluorescent antibody against SEC31A (green) and SEC12 (magenta). *Inset* of the merged image is magnified 5× (*Lower Right*). (Scale bars, 10 μm.) (*E*) Confocal immunofluorescence images of SEC12 (cyan), PC1 (yellow), and SEC16A (magenta) in Saos-2 cells. Arrowheads point to examples of large SEC12 puncta that colocalized with PC1 but not SEC16A. (Scale bars, 10 μm.)

We hypothesize that the lateral organization of SEC12 may be controlled to allow the recruitment of SAR1-GTP onto large COPII carriers, where it may serve to sustain the polymerization of the coat onto an enlarged surface (2). To test this hypothesis, we devised a fractionation scheme to separate large and regular COPII carriers generated by the cell-free reaction (Fig. 4*A*). After the incubation was completed, cell-free reactions were centrifuged for 10 min at 7,000 × *g* to sediment donor membranes. The supernatant fraction was taken and further sedimented through a step OptiPrep density gradient at 250,000 × *g* for 1 h to separate COPII carriers of regular and large cargo. Fractions taken after sedimentation were used as input for flotation, which would separate the membrane from soluble components, and the floated sedimentation fractions were analyzed by immunoblot (Fig. 4*B*). Most PC1 sedimented to the interphase between 0% and 7.5% OptiPrep in fraction 2, a relatively low buoyant density position in relation to typical COPII vesicles. In contrast, most regular COPII markers ERGIC53 and SEC22B sedimented to the interphase between 7.5% and 18% OptiPrep, fraction 4, a more typical high buoyant density position (11). Thus, the physical properties of PC1-containing and regular cargo COPII vesicles appear to differ.

To test whether TANGO1, cTAGE5, and SEC12 are packaged into COPII-coated PC1 carriers, we used immunoblot to detect these proteins in the relevant buoyant density fractions. Cofractionation of TANGO1, cTAGE5, and SEC12 with large PC1 carriers was observed, as they were more abundant at the 0–7.5% interphase (Fig. 4*C*). When the SAR1B H79G mutant was supplemented to inhibit COPII budding, less PC1 and HSP47 were detected at the 0–7.5% interphase. The enrichment of TANGO1, cTAGE5, and SEC12 in this fraction was also sensitive to the SAR1B H79G mutant, confirming that TANGO1, cTAGE5, and SEC12 were incorporated into low buoyant density COPII-coated PC1 carriers in a manner dependent upon GTP hydrolysis by SAR1 (Fig. 4*C* and *SI Appendix*, Fig. S2).

Previously, we found that small COPII vesicles were about 10- to 20-fold more prevalent than large COPII-coated PC1 carriers in the 7,000 × *g* supernatant fraction as quantified by flow cytometry and nanoparticle tracking analysis (11). Although the immunoblot in Fig. 4*C* suggested that SEC12 was only somewhat enriched in the low vs. the high buoyant density membranes, the relative enrichment per COPII vesicle may be substantially greater. We examined the localization of SEC12 in KI6 cells after 7.5 h of induced overexpression of KLHL12, conditions that produce large COPII-coated PC1 carriers, which were well-separated from the ERES marker SEC16A and resolved by STORM (11). Using confocal microscopy, we observed large SEC12 puncta that colocalized with large SEC31A puncta (Fig. 4*D*). Smaller SEC12 puncta, possibly representing ERES for regular cargo, were also observed at lower signal intensity (18) (Fig. 4*D*, *Inset*). Small SEC31A puncta represent both ERES and small COPII vesicles. Populations of small SEC31A puncta that did not colocalize with SEC12 were observed, possibly representing free small COPII vesicles that excluded SEC12 (Fig. 4*D*, *Inset*). Large SEC12 puncta were also observed by confocal microscopy in PC1-secreting Saos-2 cells not overexpressing KLHL12 (Fig. 4*E*). Because mammalian SEC12 is known to localize at the ERES, we also included the scaffold protein SEC16A as a marker for ERES. To stimulate ER export of PC1, we treated Saos-2 cells with ascorbate for 30 min before fixation. Ascorbate is a cofactor for prolyl-hydroxylase, which is required for PC trimerization, thus its addition stimulates PC1 secretion. We observed large and densely labeled SEC12 puncta that were predominantly positive for PC1, and many of these large SEC12 puncta did not colocalize with SEC16A, suggesting they were free large COPII carriers of PC1 (Fig. 4*E*, arrowheads). Large SEC12 puncta that were positive for both PC1 and SEC16A were also observed, suggesting that SEC12 also localized to PC1-containing ERES.

### SEC12 Is Localized Around PC1 in Large COPII Structures.

**Fig. 5.**
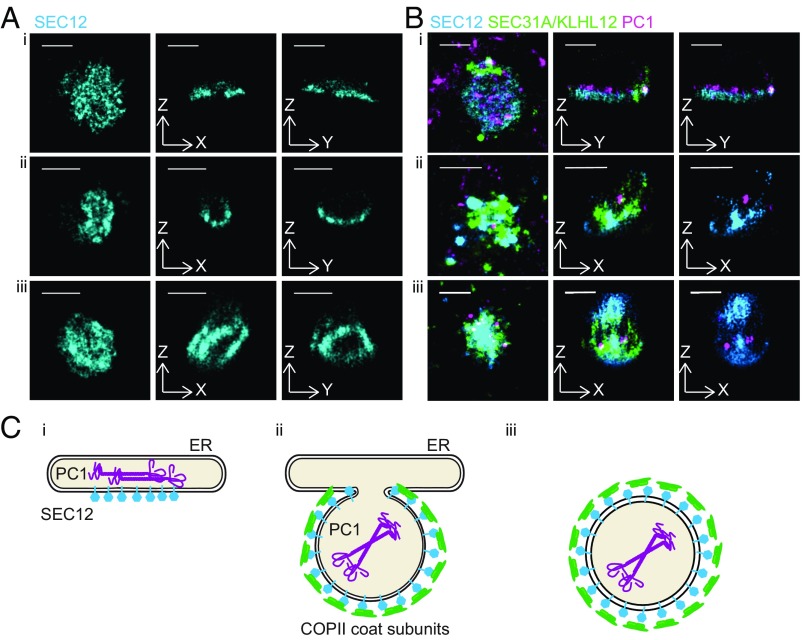
SEC12 is localized around PC1 throughout large COPII vesicle formation. (*A*) Three-dimensional (3D) STORM images of single color large SEC12 (cyan) puncta collected from Saos-2 cells. Three representative examples from three classes of ultrastructures (*i*–*iii*) are shown in magnified maximum *xy* projection (*Left*), virtual cross-sections in *xz* (*Center*), and *yz* (*Right*). (*B*) Three-color 3D STORM images of large COPII structures in KI6 cells following 7.5-h induced overexpression of KLHL12: SEC12 (cyan), PC1 (magenta), and COPII coat subunit SEC31A (green in *B*, *i* and *iii*) or KLHL12 (green in *B*, *ii*). Representative examples of three classes of ultrastructures (*i*–*iii*) are shown in three-color merged maximum *xy* projection (*Left*), three-color merged virtual cross-sections (*Center*), and two-color merged virtual cross-sections of SEC12 (cyan) and PC1 (magenta) (*Right*). (*C*) Schematic illustrations of three classes of ultrastructures of SEC12 arranged in putative time progression: (*i*) enrichment of SEC12 (cyan) around PC1 (magenta) containing ER; (*ii*) nascent large COPII (green) budding event where SEC12 localizes around membrane-containing PC1; (*iii*) free large COPII-coated PC1 carrier with enriched SEC12. (Scale bars, 500 nm.)

We next employed 3D-STORM to resolve the large SEC12 puncta observed by confocal microscopy. Three classes of ultrastructures were revealed when large SEC12 puncta over 300 nm in diameter were examined in 3D (Fig. 5*A*). The first class of large SEC12 structures were hollow spheres, similar to what we previously observed for coat component SEC31A in large COPII carriers (11) (Fig. 5 *A*, *iii*). To study the location of PC1 and the COPII coat with respect to SEC12, we performed three-color 3D STORM imaging on large SEC12/PC1/SEC31A puncta (Fig. 5 *B*, *iii*). PC1 was resolved to be inside of hollow cavities and entirely encapsulated by SEC12 and SEC31A, suggesting that these SEC12 hollow spheres were large COPII-coated PC1 carriers (Fig. 5 *C*, *iii*). The second class of large SEC12 structures were cup-shaped structures (Fig. 5 *A*, *ii*), which were also previously reported with SEC31A (11). These structures appeared to be nascent budding events at the ERES, as the cup-shaped SEC12/KLHL12 colocalized structure only partially enveloped PC1 (Fig. 5 *B*, *ii* and *C*, *ii*). A third class of large SEC12 structures appeared to be flat discs with little curvature (Fig. 5 *A*, *i*), which were not observed when the localization of the COPII outer coat protein SEC31A was analyzed by 3D-STORM. Although these SEC12 flat discs colocalized with PC1 without a discernible pattern in maximum *xy* projections, PC1 localized to only one side of the SEC12 flat discs when the 3D structure was examined (Fig. 5 *B*, *i*). The SEC12/PC1 flat discs possibly represented PC1-containing ERES before the recruitment and activation of SAR1 (Fig. 5 *C*, *i*), which would explain why little SEC31A was observed overlapping with SEC12, given that the SEC13/31 outer coat is recruited after the activation of SAR1 and the recruitment of the inner coat (28). These 3D-STORM data supported our biochemical analyses of large COPII-coated PC1 carriers generated in a cell-free reaction.

We deduced a putative temporal progression of large COPII-coated PC1 carrier formation based on the three classes of SEC12 ultrastructures revealed by 3D-STORM (Fig. 5*C*). In this speculative timeline, the concentrated targeting of SEC12 to PC1-containing ERES precedes the recruitment of the SAR1 GTPase, which is activated when SEC12 catalyzes its guanine nucleotide exchange (29–31). Because binding of SAR1 to GTP exposes an amphipathic helix that is sufficient to induce membrane curvature and recruit downstream COPII coat subunits to complete vesicle budding, the flat discs of SEC12 may be formed before the initiation of curvature (4, 32).

### Active Targeting of SEC12 to Large Cargo Increases COPII Size.

**Fig. 6.**
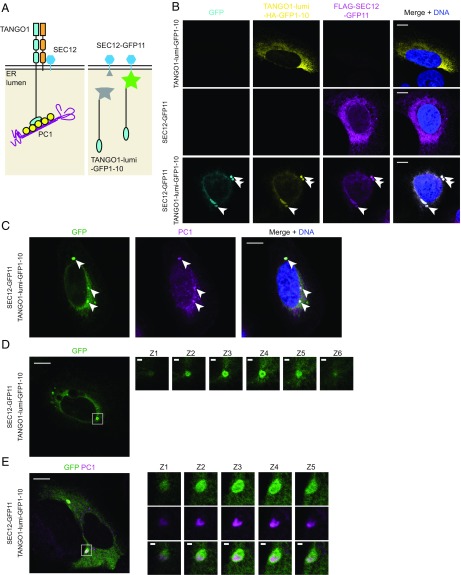
Active targeting of SEC12 to large cargo increases COPII size. (*A*) Drawings that represent the working model of SEC12 enrichment (*Left*) and the design of split GFP constructs (*Right*). In our working model, the luminal SH3 domain of TANGO1 targets SEC12 to PC1. To recapitulate sorting of SEC12 to PC1, we constructed 3xFLAG-SEC12-GFP11 and TANGO1-lumi-HA-GFP1-10 fusion constructs, where TANGO1-lumi contains TANGO1’s cargo-sensing SH3 domain. (*B*) GFP complementation brought SEC12 and TANGO1-lumi together. Confocal images of U-2OS-wt-c11 cells that were transfected with TANGO1-lumi-HA-GFP1-10 alone (*Top*), 3xFLAG-SEC12-GFP11 alone (*Middle*), or both (*Bottom*), complemented GFP (cyan), TANGO1-lumi-HA-GFP1-10 (yellow, IF against HA), and 3xFLAG-SEC12-GFP11 (magenta, IF against FLAG). (*C*–*E*) Confocal images of U-2OS-wt-c11 cells transfected with both TANGO1-lumi-HA-GFP1-10 and 3xFLAG-SEC12-GFP11 Cells were immunofluorescently labeled against PC1 (magenta) in *C* and *E* and imaged in combination with complemented GFP (green). (*D* and *E*) Cells were cultured in dialyzed medium, which contained minimal ascorbate. Ascorbate was added to cells for 30 min to stimulate PC1 export. Confocal *z*-stacks of magnified *Insets* show hollow spherical complement GFP (green) structures that entirely enveloped PC1 (magenta). Confocal *z*-stack was taken with a step size of 0.38 μm. [Scale bars: 10 μm (*B*–*E*) and 1 μm (magnified *Insets* in *D* and *E*).]

To test whether the active sorting of SEC12 could control the size of COPII carriers, we recapitulated the targeting of SEC12 to the PC1-containing ER membrane in cultured cells. Previous reports showed that TANGO1 and cTAGE5 mediate the targeting of SEC12 to ERES, as knocking down either TANGO1 or cTAGE5 resulted in diffuse ER localization of SEC12 (18, 19, 33). We considered the possibility that SEC12 is targeted to PC1-containing ERES mediated by the luminal SH3 domain of TANGO1. This SH3 domain is the only element in the TANGO1/cTAGE5/SEC12 complex that is known to bind PC1 through its interaction with HSP47 (Figs. 1*A* and 6*A*). To test this hypothesis, we employed a split-GFP targeting and detection scheme based on the self-assembly of complementary fragments of GFP fused to each target protein (34). This method has been shown to drive interaction and to visually localize targets in close proximity. Briefly, GFP11 (the 11th β-strand of GFP) was fused to the C terminus of 3xFLAG-SEC12 so that it would be exposed on the short ER luminal tail of SEC12; GFP1-10 (the rest of GFP without the 11th β-strand) was fused to the C terminus of the luminal SH3 and unstructured domains of TANGO1 (TANGO1-lumi), and an HA tag was used as linker between TANGO1-lumi and GFP1-10 (Fig. 6*A*). When transfected alone, neither construct produced GFP fluorescence and both showed ER localization, as expected (Fig. 6*B*). In cells transfected with both 3xFLAG-SEC12-GFP11 (referred to as SEC12-GFP11) and TANGO1-lumi-HA-GFP1-10 (referred to as TANGO1-lumi-GFP1-10), SEC12-GFP11 was recruited to TANGO1-lumi-GFP1-10 by GFP complementation (Fig. 6*B*). To test whether SEC12-GFP11 was targeted to PC1 when complemented with TANGO1-lumi-GFP1-10, we labeled endogenous PC1 by immunofluorescence and observed colocalization of PC1 with the complemented GFP (Fig. 6*C*). Complemented GFP signals were often ER-localized and large GFP puncta were found in a subpopulation of GFP^+^ cells (Fig. 6 *B* and *C*, arrowheads).

We used ascorbate treatment to synchronize the traffic of PC in these cells. Because regular culture medium contains a trace amount of ascorbate, we cultured cells in dialyzed medium with a minimum level of ascorbate before transient transfection with the split GFP constructs. After 20–30 min of ascorbate treatment, large GFP structures were observed, many large enough to be visualized as hollow spheres by confocal microscopy (Fig. 6*D*). GFP spheres also encapsulated endogenous PC1 as detected by immunofluorescence (Fig. 6*E*). The split-GFP–induced large COPII carriers were sometimes large enough to be resolved by confocal microscopy (*z*-stack of magnified *Insets*, Fig. 6 *D* and *E*) and appeared much larger than most endogenous carriers (Fig. 4*E*). Thus, ectopically targeting overexpressed SEC12 to PC1 further increased the size of large COPII carriers, consistent with our hypothesis that the localization of SEC12 may guide the growth of the coat (3).

We next used an antibody that detects SAR1-GTP to visualize localization to the COPII carriers (35). When wild-type SEC12-GFP11 and TANGO1-lumi-GFP1-10 were transfected together, intense labeling of large SAR1-GTP puncta was observed to colocalize with GFP puncta (*SI Appendix*, Fig. S3). In contrast, SAR1-GTP was not found at GFP puncta when the SEC12 guanine nucleotide exchange factor-deficient mutant I41A was targeted to PC1 by TANGO1-lumi (3, 36) (*SI Appendix*, Fig. S3). We also observed large SEC31A puncta that colocalized with complemented GFP and PC1, albeit at a low frequency (*SI Appendix*, Fig. S4). These data suggest that active targeting of SEC12 to PC1 can increase the size of COPII vesicles and thus facilitate in the formation of large coated vesicles.

## Discussion

As an essential transport vehicle of the early secretory pathway, COPII vesicles are of strikingly uniform and small size. In search of a mechanism to explain the genetic requirement for COPII to secrete large cargo-like PC1 (9, 10), we had previously discovered the existence of large COPII vesicles and established them as bona fide carriers of PC1 (11, 12). In this work, we examined their molecular composition and discovered that TANGO1, cTAGE5, and SEC12 are copackaged with PC1 into COPII carriers (Figs. 1 and 4). The ER export of TANGO1 was further supported by the detection of TANGO1 in large COPII-coated carriers in cultured cells as visualized by STORM and the elucidation of a recycling pathway (Figs. 1–3). Furthermore, we provide evidence to suggest that TANGO1 may mediate the sorting of SEC12 to PC1-containing ERES as a mechanism to increase the size of COPII carriers (Figs. 4–6).

COPII vesicle formation is a coordinated process centered around the guanine nucleotide status of the small GTPase SAR1, and related to this, several reported mechanisms of COPII size regulation are also linked to the guanine nucleotide status of SAR1 (10, 37). As the first step of COPII formation, SAR1 is recruited to the ER membrane by its guanine nucleotide exchange factor SEC12 (29, 30), and the subsequent nucleotide exchange exposes an amphipathic helix, which intercalates into the ER membrane, thereby inducing membrane curvature (3, 4). Activated SAR1-GTP recruits the inner coat proteins SEC23/24, where SEC23 is the GTPase activating protein (GAP) for SAR1 (28). The inner coat proteins then recruit the outer coat proteins SEC13/31, where SEC31 acts to stimulate the GAP activity of SEC23 a further 10-fold (5, 28). Regulating the local concentration of SAR1-GTP could change the timing of membrane scission and vesicle size. This is supported by the discovery of Sec24p-m11, a mutant that augments Sar1-GTPase hydrolysis and generates smaller-than-normal COPII vesicles (37). Moreover, a mechanistic study of cranio-lenticulo-sutural dysplasia, a disease caused by a deficiency in procollagen export, revealed that the mutation SEC23A M702V inhibits PC secretion by speeding GTP hydrolysis (10).

The large transmembrane protein TANGO1 is essential for the secretion of large cargo, including members of the collagen family (14, 15, 38). ER export of TANGO1 was examined previously using a cell-free reaction that supported the generation and detection of small COPII vesicles (14). Because TANGO1 was not detected in isolated COPII vesicles, the idea emerged that TANGO1 served to package but not to accompany collagen out of the ER, thus not serving the traditional role of a stoichiometric sorting receptor (14). However, the capture of the large cargo into COPII carriers was not probed in early studies; hence, a mechanism of sorting mediated by TANGO1 remained elusive (14). Recently, we reported an improved method that allowed the detection of large COPII-coated PC1 carriers generated in a cell-free reaction (11, 22). In the present study, using this approach, we demonstrated the COPII-dependent copackaging of TANGO1 with PC1 into COPII carriers (Fig. 1*C*). This result was confirmed by the detection of TANGO1 on large COPII structures in PC1-secreting cells as visualized by STORM (Fig. 1*D*) and the COPI-dependent recycling of TANGO1 (Figs. 2 and 3). Consistent with the trafficking defect observed in cells depleted of COPI (Fig. 3), a recent study identified loss-of-function mutations in the *ARCN1* gene, which encodes the subunit δ of COPI and causes a human craniofacial syndrome (39). This genetic disease showed similar symptoms to cranio-lenticulo-sutural dysplasia and osteogenesis imperfecta, which are collagen deposition diseases caused by mutations in COPII (9, 39–41).

Similar to TANGO1, the COPII initiating factor SEC12 is not detected in small COPII vesicles generated in cell-free reactions (2, 3). Although the yeast homolog Sec12p is observed to escape the ER and is returned by Rer1p in COPI vesicles, most Sec12p remains in the ER in an *rer1*-null mutant, suggesting that the escaped Sec12p accounts for only a small fraction of the total amount of Sec12p in the cell (42, 43). Recently, a truncated recombinant yeast Sec12p missing the luminal domain but retaining cytosolic and transmembrane domains was reconstituted into planar lipid bilayers with minimal yeast COPII components (Sar1p, Sec23p/24p, and Sec13p/31p), and GTP or GMP-PNP (27). This truncated Sec12p resembles mammalian SEC12 homologs, which have short luminal tails, was excluded from cargo-containing COPII buds independent of Sar1p-GTP hydrolysis (27). This sorting is likely the result of kinetic segregation from the tightly packed cargo and coat components in COPII buds, analogous to CD45 exclusion from the immunological synapse between T cells and antigen-presenting cells (44). In contrast, here we demonstrate specific enrichment of SEC12 in large COPII carriers and PC1-containing ERES (Figs. 1*C*, 4, and 5). Because CD45 on T cells can overcome kinetic exclusion by interacting with a binding partner on the antigen-presenting cells (44), the specific enrichment of SEC12 may be achieved by forming a stable complex with cTAGE5 and TANGO1 (19), where TANGO1 targets the complex to PC1 via its luminal SH3 domain (Fig. 6*A*) (14, 16). We tested this possibility by targeting SEC12 to the SH3 domain of TANGO1 using a split-GFP system and observed enrichment of SEC12 around PC1-containing ER membrane and large COPII-coated PC1 carriers (Fig. 6).

To test whether active sorting of SEC12 can lead to an increase in COPII size, we used the split GFP system to reinforce the concentration of SEC12 around PC1-containing ER membrane and observed large COPII-coated PC1 carriers as visualized by confocal microscopy (Fig. 6). The ability of SEC12 to increase vesicle size is likely a result of its catalysis of nucleotide exchange on SAR1, as high levels of SAR1-GTP were detected around complemented GFP signals at ERES (*SI Appendix*, Fig. S3). The importance of a high concentration of activated SAR1 during large cargo secretion was demonstrated in another recent report, where overexpression of wild-type SAR1 rescued secretion of collagen VII in cells where the localization of SEC12 was dispersed as a result of cTAGE5 knockdown (45).

In our split GFP system, we reconstituted the active targeting of exogenously overexpressed SEC12 with the luminal domain of TANGO1 and demonstrated the importance of TANGO1-mediated sorting of SEC12 during the generation of large COPII carriers of PC1 (Fig. 6). This overexpression system results in an abundance of SEC12 around large cargo-containing ER well above the endogenous level and thus formation of larger than normal COPII carriers of PC1. Under normal circumstances, the endogenous enrichment of SEC12 mediated by TANGO1 may be achieved by stoichiometric interaction within the TANGO1/cTAGE5/SEC12 complex. One TANGO1 molecule appears to interact with multiple cTAGE5 molecules, which in turn may recruit equivalent amounts of SEC12 (19). Thus, SEC12 may be enriched in several-fold molar excess in relation to TANGO1 around ER membrane containing folded PC1.

The cytosolic domains of TANGO1 deleted in our minimal targeting system have also been characterized for their functions during the ER export of PCs. The interaction between the cytosolic PRD domain of TANGO1 and the COPII inner coat subunit SEC23 is proposed to promote the assembly of additional inner coat arrays and stall the recruitment of the outer coat (14, 20). The cytosolic domains of TANGO1 also mediate the formation of TANGO1 rings that are proposed to encircle the necks of budding membranes (46–48). The ring structure is thought to be important for PC secretion and the morphology of the ER and Golgi apparatus.

Sedlin is a small cytosolic protein that interacts with TANGO1 and is required for PC secretion by promoting membrane scission (35). We speculate that the abundance of TANGO1 at the budding neck will recruit more Sedlin to the neck region. Sedlin preferentially binds to SAR1-GTP, which stimulates the dissociation of SAR1-GTP from the ER membrane. In light of our discovery that SEC12 extensively covered PC1-rich budding membrane (Fig. 5), membrane scission at the budding neck would be specifically necessary due to the inhibitory effect of SEC12 on vesicle fission (3). We suspect Sedlin is more dispensable for small COPII budding, as SEC12 is excluded from the prebudding complex.

The COPII outer coat subunit SEC31A is also implicated in the regulation of COPII size and PC1 secretion. SEC31A is a monoubiquitylation substrate of the E3 ubiquitin ligase CUL3, the substrate adaptor KLHL12, and cofactors PEF1 and ALG2 (12, 49). The interaction between SEC31A and KLH12 is important for the monoubiquitylation of SEC31A, enlargement of COPII, and accelerated PC1 secretion (12, 49). Compared with the necessity of TANGO1 during collagen secretion, the KLHL12–SEC31A interaction seems to be more pertinent when timely collagen secretion is required at specific stages during development. As examples, KLHL12 expression was up-regulated in embryonic stem cells (12), at specific developmental stages via the UPR transducer BBF2H7 (50), or as a result of CXCL12/CXCR signaling (51). Large COPII vesicles induced by KLHL12 overexpression often exceeded 300 nm and appeared less dependent on large cargo, as they can be induced in HEK293T cells that do not express bulky PCs (12). The potential excess of space and sacrifice in selectivity may be beneficial in the interest of speed.

In the present study, we propose a speculative model for the enlargement of COPII-coated vesicles and PC capture coordinated by TANGO1. TANGO1 interacts with HSP47 to detect the presence of folded PC trimers in the ER lumen, and recruits cTAGE5 oligomer, which would then enrich SEC12 to the PC1-containing membrane. The concentrated targeting of SEC12 may then promote the formation of larger-than-normal COPII coats by virtue of a persistent recharging of SAR1 on the coated membrane surface. Sorting of PC would thus be ensured by the copackaging of its adaptor TANGO1 into COPII vesicles, and continued rounds of sorting sustained by recycling the TANGO1–HSP47 complex back to the ERES in COPI vesicles. Although we focused on the large cargo PC1 in the present study, our finding may be broadly applicable to the secretion of other types of PCs, as TANGO1 can sense a variety of PCs through HSP47 (16).

## Materials and Methods

Detailed materials and methods describing the cell lines, plasmids, antibodies used in this study, and all experimental procedures, including in vitro budding reactions, confocal and STORM imaging analysis, are available in *SI Appendix*.

## Supplementary Material

Supplementary File
