# Supplementary material for: TANGO1 and SEC12 are copackaged with procollagen I to facilitate the generation of large COPII carriers
Source: Proc Natl Acad Sci U S A. 2018 Dec 13;115(52):E12255–64. doi: 10.1073/pnas.1814810115 (PMC6310809; doi:10.1073/pnas.1814810115)
Supplement: Supplementary File [file pnas.1814810115.sapp.pdf]

PNAS
www.pnas.org

Supplementary Information for

TANGO1 and SEC12 are co-packaged with procollagen I to facilitate the generation of large COPII carriers

Lin Yuan, Samuel J Kenny, Juliet Hemmati, Ke Xu, and Randy Schekman

Randy Schekman
Email: schekman@berkeley.edu

This PDF file includes:

Supplementary text
Materials and Methods
Figs. S1 to S4
References for SI reference citations

www.pnas.org/cgi/doi/10.1073/pnas.1814810115

Supplementary Information Appendix

SI Materials and Methods

Plasmids

Human TANOG1-FLAG in pcDNA3.1 was a gift from Vivek Malhotra lab (CRG-Centre de Regulacio Genomica, Barcelona, Spain). The split GFP plasmids pcDNA3.1 GFP1-10/GFP11 were purchased from AddGene (Cambridge, MA). TANGO1-lumi-HA-GFP1-10 was generated by cloning the luminal domain (1-1142 aa) followed by an HA tag and a linker to the N-terminus of GFP1-10 on pcDNA3.1 GFP1-10. To improve fluorescence intensity of the complemented GFP, we deleted 113-600aa from the luminal domain of TANGO1 with site-directed mutagenesis and used it as TANGO1-lumi-HA-GFP1-10 in this study. Besides improved GFP intensity, results observed using TANGO1-lumi-HA-GFP1-10 with a shortened unstructured domain were indistinguishable from the construct that contained the full-length luminal domain. Human SEC12 in p3xFLAG CMV10 plasmid was a gift from the Kota Saito lab (University of Tokyo, Tokyo, Japan). 3xFLAG-SEC12 was cloned to the N-terminus of GFP11 in pcDNA3.1 GFP11. Site-directed mutagenesis was used to introduce I41A and N43A mutations. HSP47ΔRDEL was cloned from cDNA prepared from U-2OS cells with primers "ATGCGCTCCCTCCTG" and "CATCTTGTCACCCTTAGG" into a pcDNA4 plasmid containing C-terminal strepII tag. The pcDNA4 StrepII plasmid was a gift from the Nevan Krogan lab (UCSF, San Francisco, CA).

Cell culture, transfection, drug treatment

Human lung fibroblasts IMR-90 and svIMR-90 (IMR-90 immortalized with SV-40) were obtained from Coriell Cell Repositories at the National Institute on Aging, Coriell Institute for Medical Research. Human osteosarcoma Saos-2 and U-2OS and human fibrosarcoma HT-1080 were obtained from ATCC. Because only a low percentage of U-2OS cells express endogenous PC1, we clonally selected U-2OS cells for high endogenous PC1 expression as detected by immunoblotting. The high PC1 expressing clones "wt-C11" and "wt-G5" were used in this work. IMR-90, svIMR-90, Saos-2, U-2OS-wt-C11/G5, and HT-1080 were maintained in DMEM plus 10% FBS (GE Healthcare, Chicago, IL). Dialyzed FBS (Thermo Fisher Scientific, Grand Island, NY) was used to supplement DMEM to make a dialyzed medium. The construction and maintenance of HTPC1.1 and KI6 were described in Jin et al., 2012 and Gorur et al., 2017**(1, 2)**. HTPC1.1 that stably expresses COL1A1 was constructed from HT-1080, and the doxycycline-inducible KLHL12-3xFLAG stable cell line (KI6) was constructed from HTPC1.1. DNA plasmids were transfected using lipofectamine 2000 according to the manufacturer protocol. Knockdown of ARCN1 was achieved after transfecting siRNA (Qiagen) using lipofectamine RNAiMAX according to the manufacturer protocol.

Sequence of siRNA and quantification of knockdown efficiency were described in Sirkis et al., 2017**(3)**.

Immunofluorescence, immunoblotting, antibodies

Immunofluorescence (IF) for confocal microscopy and STORM and immunoblotting (IB) analyses were performed as previously described **(2, 4)**. For the split GFP experiments, coverslips were mounted in ProLong-Diamond antifade mountant (Thermo Fisher Scientific), which better preserves GFP intensities, overnight before imaging; coverslips for other confocal experiments were mounted in ProLong-Gold antifade mountant (Thermo Fisher Scientific). For immunoblotting detection of TANGO1 in reconstituted COPII vesicles, we used a transfer buffer containing 0.1% SDS. The following antibodies were used: mouse anti-PC1 (clone 42024; QED Biosciences, San Diego, CA; 1:200 for IF); rabbit anti-PC1 (LF-41, 1:5000 for IB) was a gift of Larry Fisher (NIH, Bethesda, MD); rabbit anti-SEC31A (Bethyl Laboratories, Montgomery, TX; for IF, at 1:200 for confocal and 1:2,000 for STORM); StrepMAB Chromeo488 conjugate (IBA Life Sciences, Göttingen, Germany, 1:200 for IF); mouse anti-FLAG (Thermo Fisher Scientific; 1:5,000 for IB); goat anti-FLAG (Novus Biologicals, Littleton, CO; for IF at 1:5,000); mouse anti-HSP47 (Enzo Life Sciences Farmingdale, NY; at 1:200 for IF and 1:5,000 for IB); rabbit anti-calnexin (Abcam, Cambridge, U.K., 1:500 for IF, 1:5000 for IB), rabbit anti-TANGO1 (Sigma, St. Louis, MO., 1:200 for IF, 1:1000 for IB); rabbit anti-cTAGE5 (Sigma, 1:2000 for IB); mouse anti ERGIC53 (Enzo Life Sciences, 1:200 for IF) ; mouse anti GM130 (BD Biosciences, 1:200 for IF); mouse anti Golgin97 (Invitrogen, 1:200 for IF); mouse anti SAR1-GTP (NewEast Bioscience, King of Prussia, PA, 1:100 for IF); goat anti-SEC12 (PREB) (R&D systems, Minneapolis, MN, 1:1000 for IB. Note: this was only used for vesicles purified by density gradient flotation due to a major unrelated cytosolic band); rat anti-SEC12 was purified from hybridoma clone 6B3 provided by the Kota Saito lab (University of Tokyo, Tokyo, Japan) and 1mg/ml aliquots were used 1:1000 for IB and 1:100 for IF. Rabbit anti ribophorin I, ERGIC53, and SEC22B were made in-house and they were used at 1:5000 for IB.

Confocal imaging

Confocal imaging was acquired using Zen 2010 Software on an LSM 710 confocal microscope system (ZEISS, Oberkochen, Germany) at CRL Molecular Imaging Center (UC Berkeley, RRID:SCR_012285). The objective used was Plan-Apochromat 63×, 1.4 NA oil DIC M27 (ZEISS). The excitation lines used were 405, 488, 561, and 633nm and collected sequentially with the following spectral range: DAPI (410-481nm), GFP (492-570nm), Alexa568 (570-648nm), and Cy5 (638-755nm) with a pinhole opening of 1a.u. for all channels. Single plane images were acquired at speed 6 for 2048x2048 and

averaging of 2 frames. Z stacks of large vesicles were acquired with 0.38μm steps in Z, 2x digital zoom, at speed 8 for 1024x1024 and average of 2.

STORM imaging

Dye-labeled cell samples were mounted on glass slides with a standard STORM imaging buffer consisting of 5% (w/v) glucose, 100 mM cysteamine, 0.8mg/ml glucose oxidase, and 40µg/ml catalase in a Tris-HCl buffer (pH 7.5) **(5, 6)**. Coverslips were sealed using Cytoseal 60. STORM imaging was performed on a homebuilt setup based on a modified Nikon Eclipse Ti-E inverted fluorescence microscope using a Nikon CFI Plan Apo λ 100x oil immersion objective (NA 1.45). Dye molecules were photoswitched to a dark state and imaged as they individually returned to an emitting state, using either 647 or 560nm lasers (MPB Communications, Quebec, Canada); these lasers were passed through an acousto-optic tunable filter and introduced through an optical fiber into the back focal plane of the microscope and onto the sample at intensities of ~2 kW cm^{-2}. A translation stage was used to shift the laser beams towards the edge of the objective so that light reached the sample at incident angles slightly smaller than the critical angle of the glass-water interface. A 405nm laser was used concurrently with either the 647 or 560nm lasers to reactivate fluorophores into the emitting state. The power of the 405nm laser (typical range 0-1 W cm^{-2}) was adjusted during image acquisition so that at any given instant, only a small, optically resolvable fraction of the fluorophores in the sample were in the emitting state. For 3D STORM imaging, a cylindrical lens was inserted into the imaging path so that images of single molecules were elongated in opposite directions for molecules on the proximal and distal sides of the focal plane **(5)**. The raw STORM data was analyzed according to previously described methods **(5, 6)**. Data was collected at a framerate of 110Hz, for a total of ~80,000 frames per image.

Three-color imaging was performed on targets labeled by Alexa Fluor 647, CF680, and CF568 via sequential imaging with 647nm and 560nm excitation. With 647nm excitation, a ratiometric detection scheme was employed to concurrently collect the emission of Alexa Fluor 647 and CF680 **(7, 8)**. Emission of these dyes was split into two light paths using a long pass dichroic mirror (T685lpxr; Chroma), each of which were projected onto one-half of an Andor iXon Ultra 897 EM-CCD camera. Dye assignment was performed by localizing and recording the intensity of each single molecule in each channel. Excitation (560 nm) was subsequently used to image CF568 through the reflected light path of the dichroic mirror.

Vesicle budding reaction

The PC1 budding reaction was performed as described in Gorur et al., 2017 and Yuan et al., 2017 **(2, 4)**. To separate COPII-coated PC1 carriers and regular COPII vesicles, we scaled-up budding reactions 4-fold. In a polycarbonate 11x34mm ultracentrifugation

tube (Beckman Coulter), 500µl of 18% (w/v) OptiPrep in B88 (20mM HEPES, pH 7.2, 250mM sorbitol, 150mM KOAc, 5mM $Mg(OAc)_2$) was placed at the bottom of the tube, overlayed with 400µl 7.5% (w/v) OptiPrep (Sigma-Aldrich) in B88, then 350µl of the 7,000xg supernatant of the reaction. The OptiPrep gradient was centrifuged at 250,000xg for 1h at 4°C (Beckman TLS-55) with slow acceleration and deceleration, after which 100µl fractions were collected from the top. Low buoyant density membranes concentrated in the region of 300-400µl from the top (referred to as Fraction 2 in text), and higher buoyant density membranes were collected at position 700-800µl from the top (referred to as Fraction 4 in text). Desired fractions were subjected to flotation: 85 µl of each sedimentation fraction was gently mixed with 50µl of 60% (w/v) OptiPrep until the sample was homogeneous, placed at the bottom of a 7×20 mm tube (Beckman Coulter), and overlaid with 100µl of 18% (w/v) and 10µl of 0% OptiPrep in B88. The OptiPrep gradient was centrifuged at 250,000xg for 1h at 4°C (Beckman TLS-55 with adaptors for 7×20mm tubes) with slow acceleration and deceleration, after which 40µl was collected from the top and mixed with sample buffer for immunoblotting analysis.

Tryptophan fluorescence assay

The tryptophan fluorescence assay was performed at 37°C in a stirred-cuvette as previously described with slight modifications **(9)**. To HKM buffer (20mM HEPES, pH 7.2, 160mM KOAc, 1mM $Mg(OAc)_2$) an indicated amount of WT, I41A, or N43A SEC12-cyto (cytosolic domain of SEC12) was added, followed by 2µM SAR1BΔN (the N-terminal amphipathic helix was omitted because it does not affect GTP/GDP binding) **(10)**, then 30µM GTP. The fluorescence intensity was followed for 15-20min until the exchange of GDP for GTP equilibrated as detected on a Cary Eclipse Fluorescent Spectrophotometer (Agilent Technologies, Santa Clara, CA) with the following settings: excitation wavelength 297nm; slit size 2.5nm; emission wavelength 340nm, slit size 20nm; high pmt detection (800v); fluorescence reading every 1s for 20min in kinetic mode.

Protein purification

COPII proteins were purified as described previously in Kim et al., 2005 and Fromme et al., 2007 **(11, 12)**. SAR1B WT, H79G, and SAR1BΔN were purified from E coli with glutathione beads (GE) and cleaved with thrombin. SEC23A/24D, SEC13/31A, SEC12-cyto WT, I41A, and N43A were purified form sf9 cells with Ni-NTA followed by a monoQ column.

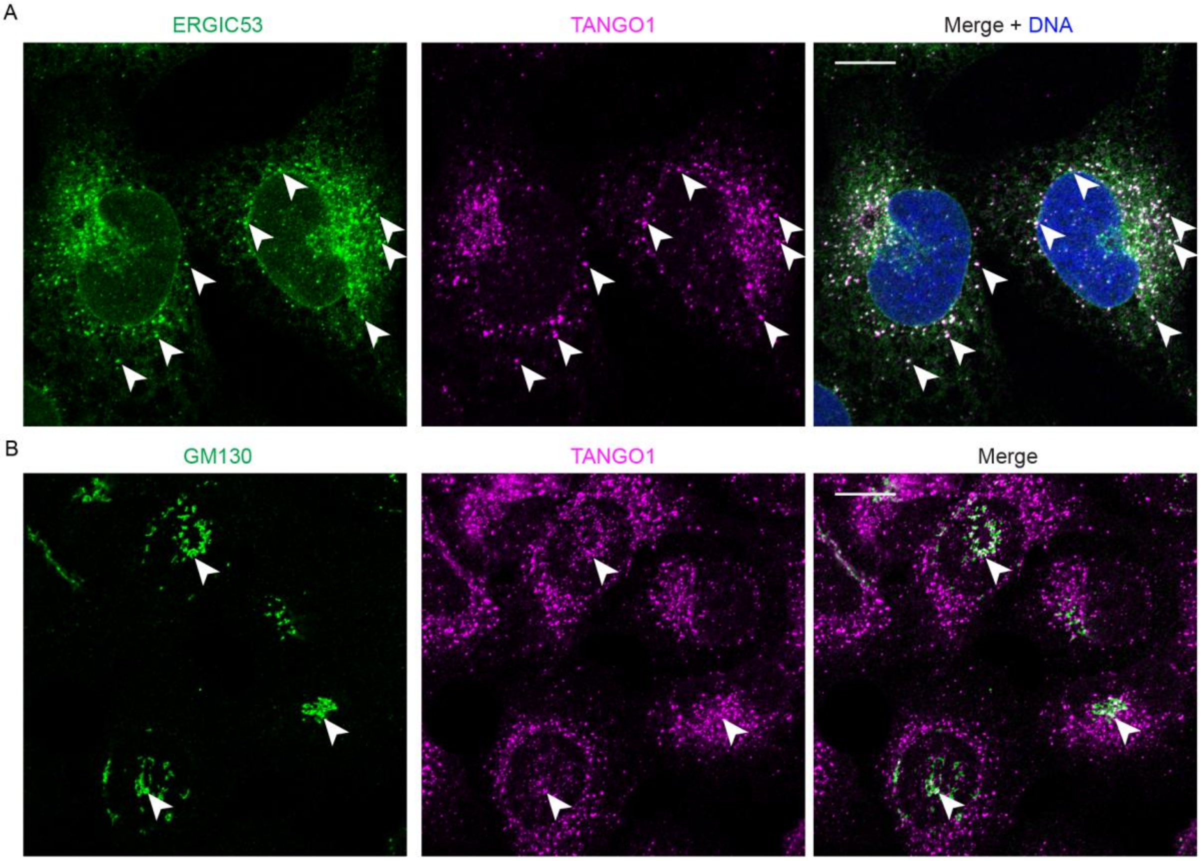

Fig. S1. TANGO1 co-localizes with ERGIC53 and GM130 at steady state. Immunofluorescent labeling of U2OS-wt-C11 cells with TANGO1 (magenta), ERGIC53 (A: green), and GM130 (B: green). TANGO1 extensively co-localized with the ERGIC marker ERGIC53 and occasionally co-localized with the cis-Golgi marker GM130 at steady state. Arrowheads point to examples of co-localization. Scale bars: 10 μm.

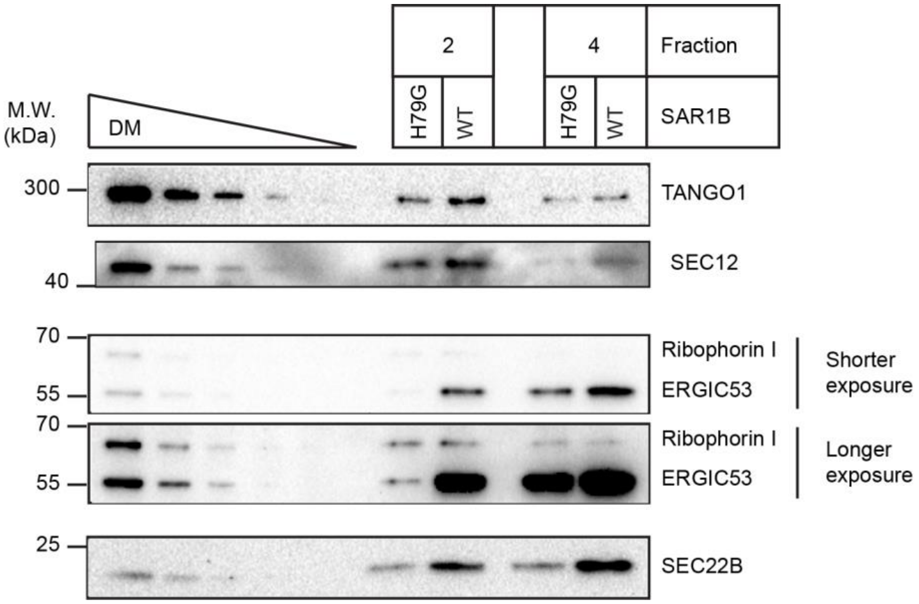

Fig. S2. ER contamination does not contribute to the TANGO1 and SEC12 signal of export in the cell-free reconstituted reaction. A closer examination of reconstituted COPII carriers prepared as described in Fig. 4 A-C. Donor membrane (DM) was loaded in the left 5 lanes (from left to right: 0.8%, 0.4%, 0.2%, 0.1%, 0.05% of input into the reactions) for comparison of immunoblot band intensities. Budding reactions were supplemented with wild-type (WT) or H79G mutant SAR1B. Buoyant membrane from relevant sedimentation fractions were immunoblotted for TANGO1 and SEC12. Ribophorin I was used as an ER marker. ERGIC53 and SEC22B served as markers for regular COPII vesicles. Consistent with Fig. 4 B-C, TANGO1 and SEC12 were enriched in the lower density interphase fraction 2, whereas ERGIC53 and SEC22B were enriched in the higher density interphase fraction 4. The export of TANGO1, SEC12, ERGIC53 and SEC22B substantially decreased in the reaction supplemented with the GTP locked SAR1B H79G mutant, showing that their export required the generation of COPII coated vesicles. In contrast, although ribophorin I was detected after longer exposure, similar amounts were observed in reactions supplemented with WT and H79G mutant. Therefore, the ER contamination does not contribute to the signal of export of TANGO1 and SEC12, and it may explain the detection of COPII cargos when SAR1B H79G was supplemented.

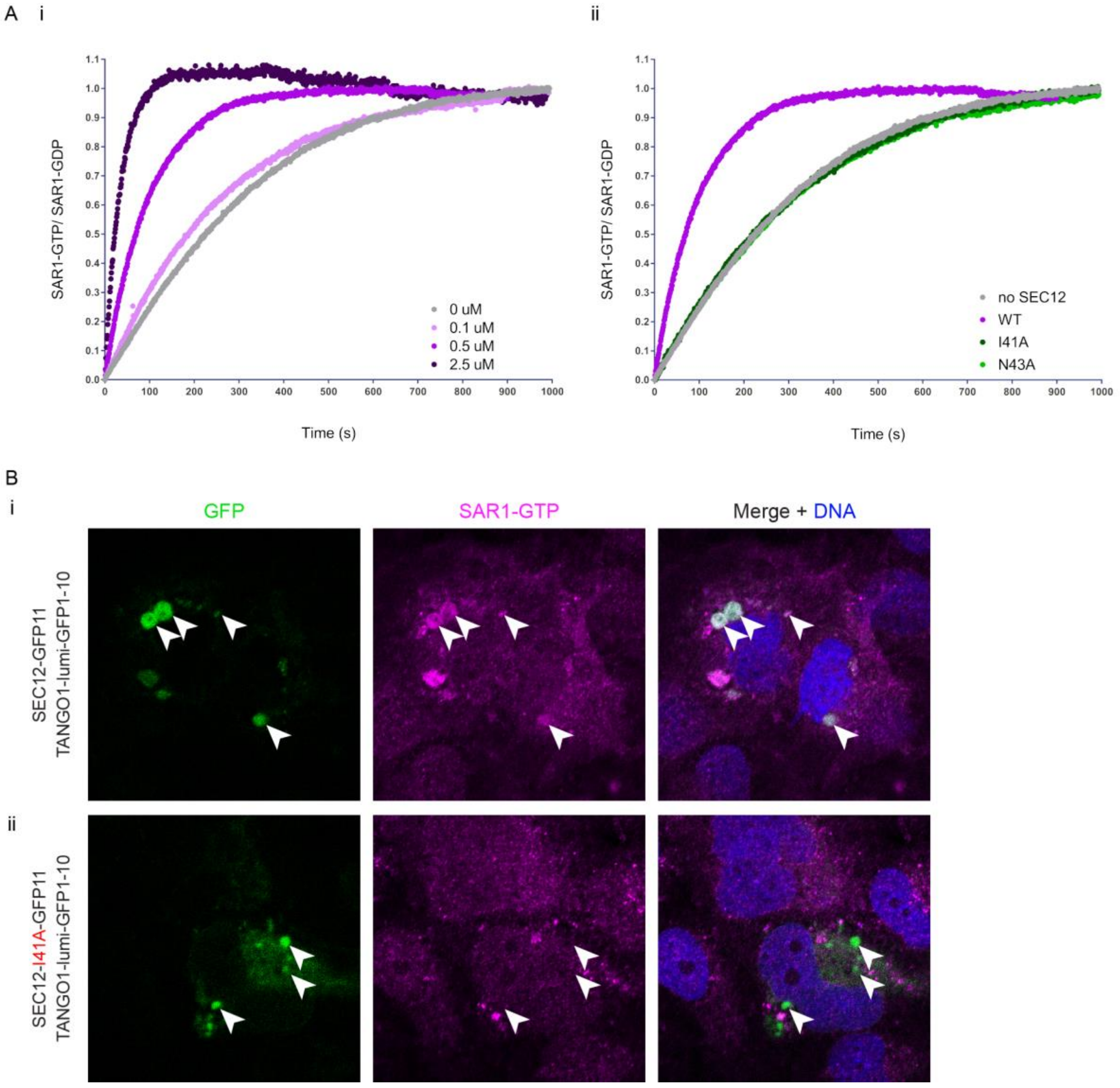

Figure S3. Complemented GFP puncta contain SEC12 with active GEF activity. (A) Mutations in I38 or N40 in Sec12p ablated its GEF activity (9, 13). Thus we mutated the conserved I41 and N43 residues in human SEC12 and tested the GEF activity of wild-type (WT), I41A, and N43A using a tryptophan fluorescence assay. Changes in tryptophan fluorescence intensity were measured with time as an indicator of the nucleotide associated with SAR1 (14). Fraction of SAR1-GTP was calculated by normalizing against the total increase of tryptophan fluorescence after GDP was exchanged to GTP. (i-ii) GDP to GTP exchange occurred naturally (grey) or in the presence of GEF deficient mutants I41A or N43A (green) or was accelerated by WT SEC12 (magenta). Indicated amounts of WT SEC12 were used in (i), and 0.5 μM of WT or mutant SEC12 were used in (ii). (B) Accumulation of SAR1-GTP (magenta) at complemented GFP (green) puncta was observed by confocal microscopy in cells expressing WT SEC12 (i) but not the GEF deficient I41A mutant (ii). U-2OS cells were transfected with both TANGO1-lumi-HA-GFP1-10 and 3xFLAG-SEC12-GFP11 (WT or

I41A as indicated), immunofluorescently labeled against SAR1-GTP, and imaged for the complement GFP and labeled SAR1-GTP.

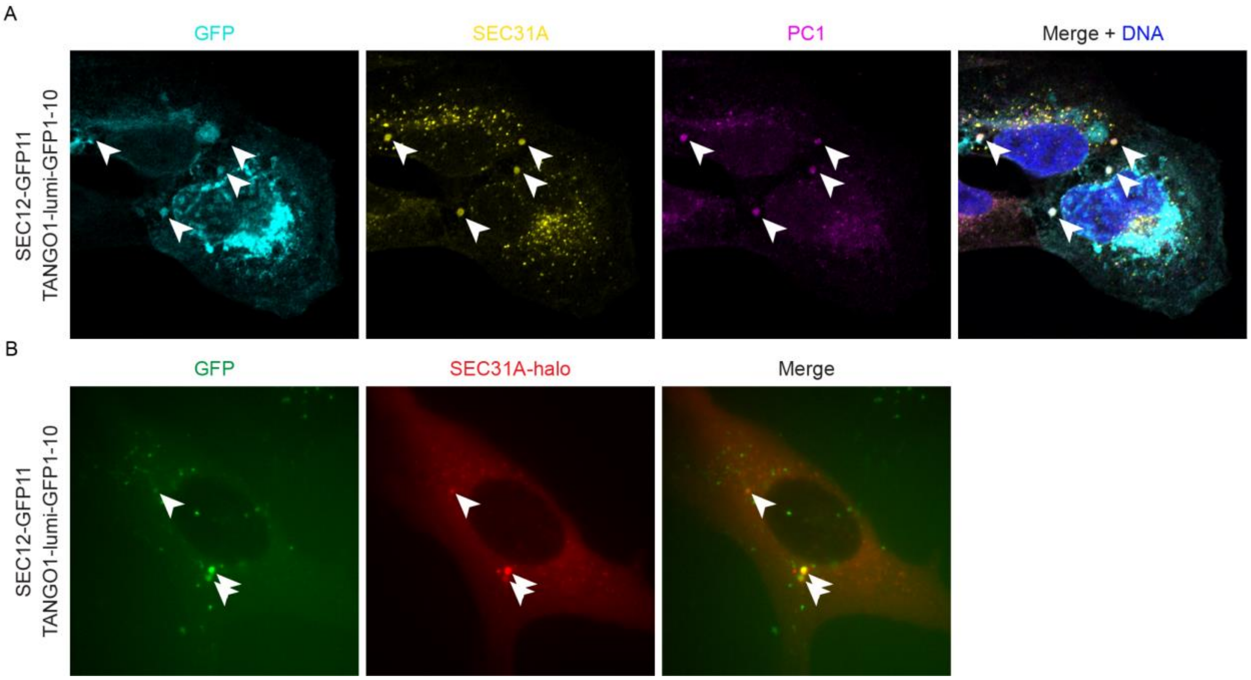

Figure S4. SEC31A colocalizes with large complemented GFP puncta. (A) U-2OS cells were transfected with both TANGO1-lumi-HA-GFP1-10 and 3xFLAG-SEC12-GFP11, then immunofluorescence was visualized by confocal microscopy with PC1 (yellow) and SEC31A (magenta) labels. Occasionally, cells containing large punctate complemented GFP (cyan) signal also show large colocalizing SEC31A and PC1 puncta. (B) U-2OS cells were transfected with SEC31A-halo in addition to TANGO1-lumi-HA-GFP1-10 and 3xFLAG-SEC12-GFP11. Live cells were imaged on an epifluorescence microscope after SEC31A-halo was labeled with the halo ligand TMR (red). Large puncta that contained complement GFP (green) and SEC31A (red) were observed at very low frequency.

References

1. Jin L, et al. (2012) Ubiquitin-dependent regulation of COPII coat size and function. *Nature* 482(7386):495–500.

2. Gorur A, et al. (2017) COPII-coated membranes function as transport carriers of intracellular procollagen I. *The Journal of cell biology*. doi:10.1083/jcb.201702135 .

3. Sirkis DW, Aparicio RE, Schekman R (2017) Neurodegeneration-associated mutant TREM2 proteins abortively cycle between the ER and ER-Golgi intermediate compartment. *Molecular biology of the cell* 28(20):2723–2733.

4. Yuan L, Baba S, Bajaj K, Schekman R (2017) Cell-free Generation of COPII-coated Procollagen I Carriers. *BIO-PROTOCOL* 7(22). doi:10.21769/bioprotoc.2450 .

5. Huang B, Wang W, Bates M, Zhuang X (2008) Three-Dimensional Super-Resolution Imaging by Stochastic Optical Reconstruction Microscopy. *Science* 319(5864):810–813.

6. Rust MJ, Bates M, Zhuang X (2006) Sub-diffraction-limit imaging by stochastic optical reconstruction microscopy (STORM). *Nat Methods* 3(10):nmeth929.

7. Bossi M, et al. (2008) Multicolor Far-Field Fluorescence Nanoscopy through Isolated Detection of Distinct Molecular Species. *Nano Letters* 8(8):2463–2468.

8. Testa I, et al. (2010) Multicolor Fluorescence Nanoscopy in Fixed and Living Cells by Exciting Conventional Fluorophores with a Single Wavelength. *Biophysical Journal* 99(8):2686–2694.

9. Futai E, Hamamoto S, Orci L, Schekman R (2004) GTP/GDP exchange by Sec12p enables COPII vesicle bud formation on synthetic liposomes. *The EMBO Journal* 23(21):4146–55.

10. Lee MC, et al. (2005) Sar1p N-terminal helix initiates membrane curvature and completes the fission of a COPII vesicle. *Cell* 122(4):605–17.

11. Kim J, Hamamoto S, Ravazzola M, Orci L, Schekman R (2005) Uncoupled Packaging of Amyloid Precursor Protein and Presenilin 1 into Coat Protein Complex II Vesicles. *Journal of Biological Chemistry* 280(9):7758–7768.

12. Fromme J, et al. (2007) The genetic basis of a craniofacial disease provides insight into COPII coat assembly. *Developmental cell* 13(5):623–34.

13. McMahon C, et al. (2012) The structure of Sec12 implicates potassium ion coordination in Sar1 activation. *Journal of Biological Chemistry* 287(52):43599–43606.

14. Antonny, Madden D, Hamamoto S, Orci L, Schekman R (2001) Dynamics of the COPII coat with GTP and stable analogues. *Nature cell biology* 3(6):531–7.
